# Anterior Cervical Corpectomy and Reconstruction With Fibular Autograft for Bacterial Spondylodiscitis: A Case Report and Literature Review

**DOI:** 10.7759/cureus.104236

**Published:** 2026-02-25

**Authors:** Wei-Hsin Chang, Jyun-Yuan Huang, Tzu-Ning Chen, Jui-Sheng Chen

**Affiliations:** 1 Department of Neurosurgery, E-Da Hospital, Kaohsiung, TWN; 2 College of Medicine, I-Shou University, Kaohsiung, TWN

**Keywords:** cervical corpectomy, cervical reconstruction, epidural abscess, fibular autograft, spondylodiscitis

## Abstract

Bacterial spondylodiscitis of the cervical spine is an uncommon but potentially serious condition that may require surgical intervention in cases of progressive vertebral collapse or neurological deterioration. We report a 41-year-old man who presented with intermittent fever, neck pain, and quadriparesis and was diagnosed with C5-C7 spondylodiscitis complicated by epidural and prevertebral abscess caused by oxacillin-sensitive *Staphylococcus aureus*. Despite two months of appropriate antibiotic therapy, follow-up imaging demonstrated progressive collapse of the C6-C7 vertebral body. The patient subsequently underwent C5-C7 corpectomy and reconstruction using a fibular autograft with anterior plating, along with posterior fixation using C4 and C5 lateral mass screws and T1 and T2 pedicle screws. Postoperatively, the patient showed significant neurological improvement, including recovery of ambulation and fine hand function. This case highlights fibular autograft as a viable and dependable reconstruction option following cervical corpectomy for bacterial spondylodiscitis.

## Introduction

Bacterial spondylodiscitis is one of the diseases that neurosurgeons least want to encounter. It is an infection of the intervertebral disc and the adjacent vertebral bodies. Patients often become bacteremic from sources such as endocarditis and intravenous drug use. The symptoms of bacterial spondylodiscitis are non-specific. While back pain or neck pain is common, 15% of patients do not experience pain. Fever is less common, occurring in only about half of the patients. Spinal deformities, predominantly kyphosis and gibbus formation, are more common in tuberculous spondylitis. Untreated chronic infections can progress to sinus formation. Cervical spondylodiscitis may manifest with dysphagia or torticollis. Spinal tenderness is the most common sign detected on examination [1].

Generally, a six-week course of antibiotic treatment is adequate for those without neurologic deficits. However, if there is a presence of neurologic deficit, failure to respond to antibiotics, or structural instability, corpectomy with reconstruction is indicated [2]. In the modern medical era, reconstruction with titanium or porous tantalum mesh cages is popular. However, these metal cages can be prohibitively expensive for some patients. Therefore, harvesting a fibular autograft remains an important skill for reconstruction.

In this report, we present a case of bacterial spondylodiscitis with a neurologic deficit. Corpectomy and reconstruction with a fibular autograft were performed, resulting in a significant improvement in the patient's postoperative neurologic condition and cervical stability.

## Case presentation

**Figure 1:**
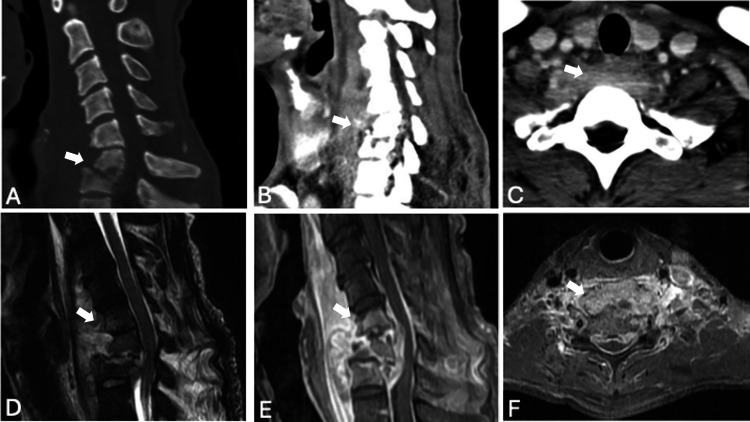
Preoperative images (A) Preoperative bone window cervical sagittal CT of the patient revealing C6-C7 bone erosion with malalignment (white arrow). (B-C) Preoperative cervical sagittal and axial CT with contrast revealing C5-C7 prevertebral abscess (white arrow). (D-F) Preoperative T2-weighted sagittal and T1-weighted sagittal and axial views with gadolinium revealing C5-C7 spondylodiscitis with epidural and prevertebral abscess (white arrow). Additionally, there is C6-C7 cord compression with signal change. CT: computed tomography

The patient is a 41-year-old man with underlying liver cirrhosis, Child-Pugh class A, chronic hepatitis C, and low socioeconomic status. Initially, he experienced neck pain, intermittent fever without chills, general malaise, and poor appetite for a week. On January 7, 2024, he suddenly developed quadriparesis and was immediately taken to our emergency department. Neurological examination revealed weaker muscle power on the right side compared to the left (the right upper limb and right lower limb were all 2, while the left upper limb was 4, and the left lower limb was 3), increased deep tendon reflexes on the right side, and pinprick hypoesthesia in the bilateral C5-C6 dermatomes. Laboratory data indicated leukocytosis with left shift, with a white blood cell count of 14,820/μL (reference range: 4,000-10,000/μL) and neutrophil predominance (segment: 92.3%; reference range: 40-75%). C-reactive protein was markedly elevated at 177.71 mg/L (reference range: <5 mg/L), and the erythrocyte sedimentation rate was 66 mm/hour (reference range: <20 mm/hour). Cervical computed tomography showed C6-C7 bone erosion with malalignment and C5-C7 prevertebral abscess (Figure 1A-1C). Cervical magnetic resonance imaging with contrast revealed C5-C7 spondylodiscitis with epidural and prevertebral abscess (Figure 1D-1F).

**Figure 2:**
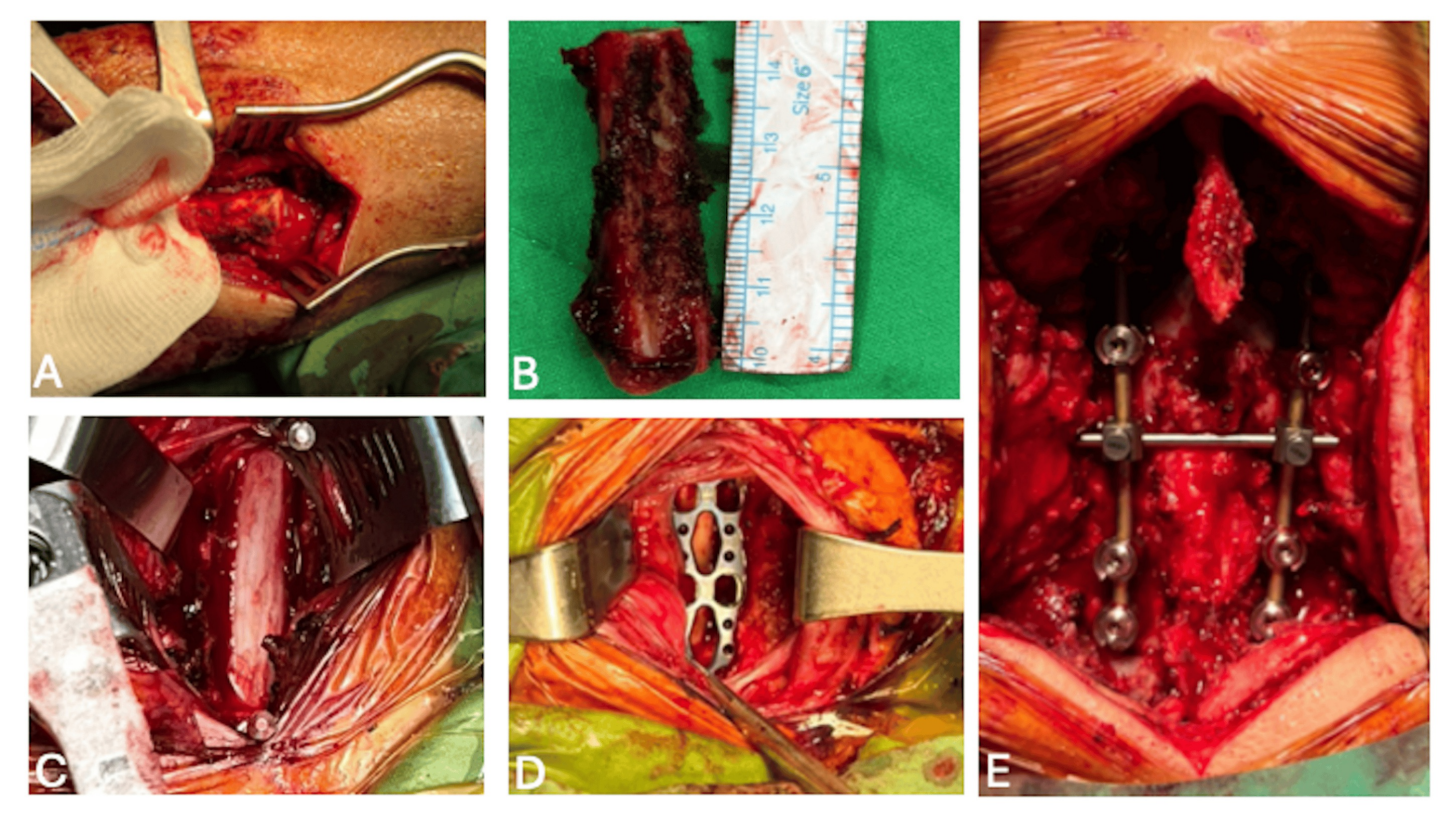
Intraoperative images (A) Harvesting autograft from the left fibular bone. The proximal cut edge is 10 cm below the fibular head. (B) The fibular autograft is 4.7 cm in length. (C-D) A 4.2 cm fibular autograft was prepared and placed into the C5-C7 corpectomy site, followed by anterior plate fixation. (E) Posterior fixation using C4-C5 lateral mass and T1-T2 transpedicular screws.

Treatment with intravenous vancomycin, ceftazidime, and metronidazole was initiated. Given the presence of neural compromise and epidural involvement, an urgent posterior decompressive laminectomy (C5-C7) was performed to relieve cord compression and facilitate infection control on January 9. This was followed by anterior debridement of the prevertebral space to reduce the infectious burden and obtain microbiological specimens. Cultures identified oxacillin-sensitive *Staphylococcus aureus*. Following the antibiotic course with oxacillin for two months, the infection parameters improved, and follow-up cervical magnetic resonance imaging showed no abscess progression but a gradually collapsed C6-C7 vertebral body. Because the patient cannot afford a metal cage mesh, a C5-C7 corpectomy and reconstruction with fibular autograft with anterior plating fixation, along with posterior fixation using C4-C5 lateral mass and T1-T2 transpedicular screws, were performed (Figure 2).

**Figure 3:**
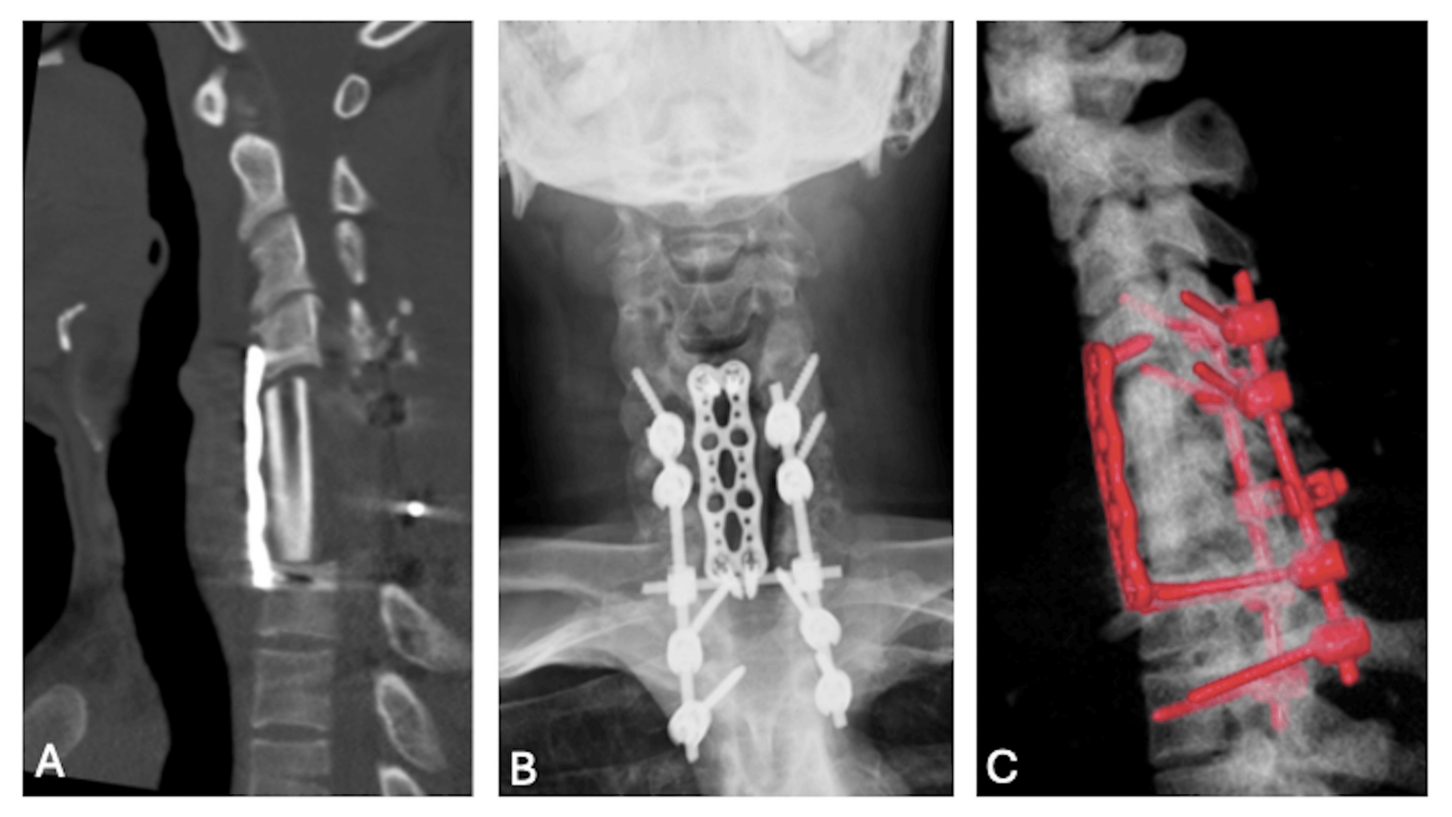
Postoperative images (A, C) Postoperative X-ray and three-dimensional reconstruction images showing the accurate positions into which the plates and screws were installed. (B) Postoperative bone window cervical sagittal CT showing fibular autograft placed in a proper position. CT: computed tomography

Postoperatively, the patient's cervical stability remained fair, and neurological function progressively improved. He regained ambulatory ability and demonstrated enhanced fine motor control in both upper extremities. The postoperative care regimen included rigid cervical collar immobilization for a total of 12 weeks, with activity initially limited to light ambulation under supervision. During the first two weeks, passive range-of-motion exercises for the shoulders and elbows were initiated, along with isometric neck stabilization in the neutral position to reduce paraspinal muscle atrophy. From postoperative weeks 3 to 8, the patient began gradual active-assisted neck movements within a pain-free range, gait training for balance and proprioception, and occupational therapy to improve hand coordination and dexterity. After postoperative week 8, more intensive cervical muscle strengthening and upper limb functional training were introduced while continuing protective collar use until the end of the 12th week. Clinical and radiographic evaluations at one, three, and six months confirmed stable graft alignment without subsidence or pseudoarthrosis. By the six-month follow-up, the patient was neurologically intact, pain-free, and fully independent in daily activities (Figure 3).

## Discussion

Spondylodiscitis is not an unusual disease, with an incidence of 5.4 per 100,000 persons per year. The most common site is the lumbar spine (58%), followed by the cervical spine (11%) [2]. Spondylodiscitis can be divided into two categories: pyogenic and non-pyogenic. The pyogenic type is usually caused by bacterial infection, whereas the non-pyogenic type is associated with tuberculosis, fungi, and parasites, for example, *Diphyllobothrium latum* or *Taenia solium* [3].

The main treatment for pyogenic spondylodiscitis is antibiotic therapy. Traditionally, the duration has been recommended to exceed six weeks. However, Li et al. reported that in patients without positive blood cultures and without paraspinal abscess, a three-week antibiotic regimen may be effective [4].

The surgical indications for spondylodiscitis include neurological deficit, vertebral instability, and poor response to conservative treatment. When corpectomy is indicated, reconstruction is required to replace the intervertebral disc and vertebral body, using materials such as autograft bone or a mesh cage. However, mesh cages remain relatively expensive. For patients who cannot afford metallic implants, autograft or allograft bone becomes an alternative option.

Fibular autograft has been widely used in orthopedic and plastic reconstructive surgery. To prevent common peroneal nerve injury, the proximal osteotomy should be performed at least 10 cm below the fibular head. To avoid ankle instability, the distal cut should be made 6-8 cm above the lateral malleolus [5]. Mukherjee et al. developed a minimally invasive technique for harvesting fibular grafts [6]. This method utilizes a special periosteal stripper and requires only two small incisions to completely free the graft. The resulting postoperative scars are cosmetically favorable.

In 1995, Jitsuhiko Shikata first introduced the use of a fibular autograft strut bone for spinal reconstruction [7]. He developed the Shikata technique, which involved shaping the upper portion of the graft into an anterior fin approximately 0.6 cm in height, along with a posterior slope designed to engage the inferior surface of the superior vertebra. After impaction of the graft into the prepared bed, a distal diagonal screw was inserted into the inferior vertebra for stabilization.

Bongers et al. utilized free vascularized fibular grafts (FVFG) for the reconstruction of the mobile spine following tumor resection [8]. FVFG proved to be an effective technique, particularly in the cervicothoracic region. The reported complete union rate was 76%. However, a notable rate of implant failure was observed in reconstructions below the L1 vertebra.

From a biomechanical perspective, the fibula provides a robust cortical bone structure that ensures sufficient axial load-bearing capacity, which is essential for spinal reconstruction. Its long, tubular morphology makes it particularly suitable for anterior column support in the cervical spine. In addition to its mechanical strength, the fibular autograft demonstrates excellent biocompatibility and osteoconductive properties, facilitating graft-host integration and long-term fusion. As an autologous graft, it eliminates the risks of immunogenic rejection and transmissible disease associated with allografts.

In a cadaveric study by Marchetto et al. [9], C5 corpectomy followed by reconstruction using a fibular graft demonstrated excellent immediate structural stability under flexion loading. None of the specimens exhibited graft fracture or extrusion during biomechanical testing, indicating that the fibula provides sufficient rigidity to maintain alignment under physiological loads. Interestingly, mechanical failure occurred predominantly at the interface between the graft and adjacent vertebral bodies, typically as fractures of the vertebral endplates rather than failure of the fibular graft itself. This finding suggests that the cortical structure of the fibula is biomechanically superior to the surrounding cancellous bone.

The average maximum flexion moment before failure was 17.73 Nm, comparable to that observed in intact spines from younger male cadavers. The fibula's dense cortical bone, high axial load-bearing capacity, and compatibility with vertebral morphology contribute to its favorable performance. Additionally, creating notches at both ends of the graft facilitates mechanical interlock with adjacent vertebrae and reduces the risk of displacement. These findings support the use of fibular autograft as a reliable and structurally competent option for anterior cervical reconstruction, particularly in settings where metallic implants are less feasible.

van den Heuvel et al. demonstrated favorable postoperative quality of life following this complex reconstructive surgery, highlighting the viability and durability of FVFG in spinal reconstruction, despite complications such as deep infections and hardware breakage [10].

Nassr et al. reported outcomes from 163 cases of autogenous non-vascularized fibular strut graft harvest for anterior cervical corpectomy and fusion. Short-term complications included incisional pain lasting up to three months (53%), cellulitis (9%), and deep infection (0.6%). These were generally managed conservatively or with antibiotics, and debridement was rarely required. Long-term complications included prolonged incisional pain, superficial peroneal neuroma (1.2%), ankle instability (1.2%), and tibial stress fracture (3%). Superficial peroneal neuroma was typically treated supportively. One case of ankle instability required Broström reconstruction, while others were managed with ankle bracing. Tibial stress fractures in two patients were treated with bracing, though healing was uneven. Cases managed with intramedullary screws achieved successful resolution, and only one additional case required free tissue transfer due to complications [11].

Subramaniam et al. conducted a retrospective review of 48 patients who underwent multilevel anterior cervical corpectomy involving three or more levels. Patients were divided into two groups based on the reconstruction method: autogenous fibular strut graft (Group A) or titanium mesh cage with rigid cervical plating (Group B) [12]. There was no significant difference in postoperative two-year Nurick scores, Modified Japanese Orthopaedic Association (mJOA) scores, cervical segmental angles, or cervical sagittal alignment between the two groups. However, Group B demonstrated a significantly higher fusion rate compared to Group A (95.8%±0.2% vs. 83.3%±0.8%; p=0.001). Additionally, Group B had a significantly lower rate of graft subsidence (2%±0.6% vs. 3%±0.8%; p=0.002).

This case report is limited by its single-patient design and lack of a control group, which restricts the generalizability of the findings. Although the clinical outcome was favorable, larger case series and comparative studies, particularly those evaluating alternative reconstruction methods such as titanium mesh cages, are warranted to further validate the effectiveness and broader applicability of fibular autografts in cervical spine reconstruction.

## Conclusions

In summary, this case demonstrates that reconstruction with fibular autograft combined with circumferential stabilization can be a feasible and effective option for managing cervical spondylodiscitis requiring corpectomy. The favorable outcome likely reflects the combined effects of thorough debridement, appropriate antimicrobial therapy, biological graft reconstruction, and mechanical stability provided by instrumentation.
